# Pulmonary sequestration and endovascular treatment: a case report

**DOI:** 10.1590/1677-5449.011018

**Published:** 2019-01-10

**Authors:** Sergio Quilici Belczak, Ingredy Tavares da Silva, Jéssica Cunha Bernardes, Felipe Basso de Macedo, Laís Leite Lucato, Bruna Rodrigues, Bruna Stecca Zeque

**Affiliations:** 1 Centro Universitário São Camilo, São Paulo, SP, Brasil.; 2 Instituto de Aprimoramento e Pesquisa em Angiorradiologia e Cirurgia Endovascular – IAPACE, São Paulo, SP, Brasil.

**Keywords:** pulmonary sequestration, therapeutic embolization, pulmonary circulation, endovascular procedures

## Abstract

Pulmonary sequestration is a congenital anomaly defined as a nonfunctioning mass of lung parenchyma. Presence of an independent pleural envelope classifies it as intralobar, accounting for approximately 75% of the cases, while absence classifies cases as extralobar, accounting for the remaining 25%. Diagnosis is made through radiography and confirmed by computed tomography, magnetic resonance, or angiography. The traditional treatment is open surgical repair, but endovascular techniques have been used, with good results. We report the case of a 29-year-old-woman presenting with recurrent pneumonia for 5 years. A CT scan of the chest revealed poor vascular formation in the lower region of the right lung. The pulmonary sequestration was treated by embolization of the anomalous branch.

## INTRODUCTION

 Although pulmonary malformations are classified as distinct pathologies, they comprise a group of anomalies with very similar clinical manifestations that have their origins in failures of embryonic development. The most common malformations include cystic adenomatoid malformation, pulmonary sequestration, congenital lobar emphysema, bronchogenic cysts, and pulmonary arteriovenous malformations. 1 Of these, pulmonary sequestration accounts for 0.15 to 6.45% of pulmonary malformation cases. 1 ^-^ 3

 Pulmonary sequestration is thus a rare congenital anomaly. It consists of a non-functional mass of pulmonary parenchyma that does not communicate with the normal tracheobronchial tree and which receives its blood supply from an anomalous systemic artery, generally from the descending aorta, although other sources of arterial supply are possible. Drainage may be via systemic veins or pulmonary veins. 1 ^-^ 3 The tissue is embryonic cystic tissue and non-aerated and disorganized alveoli, in addition to other respiratory tract components. 1

 The malformation can be classified as one of two distinct forms, intralobar or extralobar, on the basis of whether it has its own pleural envelope. 1 ^-^ 3 Intralobar pulmonary sequestration is present when the sequestrated area is surrounded by the visceral pleura of a normal pulmonary lobe. 1 ^-^ 3 This form accounts for approximately 75% of sequestration cases, and most commonly involves the inferior left lobe, with equal distribution by sex. Intralobar cases tend to have later clinical manifestation, during the second decade of life, generally with recurrent respiratory infections, hemoptysis, and dyspnea. 1 ^,^ 3

 In extralobar pulmonary sequestration, the sequestrated region is surrounded by its own pleural envelope, accounting for the remaining 25% of cases of this type of pulmonary malformation. 1 ^-^ 3 This form also predominantly involves the left lower lobe, but, in contrast with intralobar sequestration, prevalence is higher in males (4:1). 2 ^,^ 3 Clinical presentation tends to occur in the first few months of life and it is often associated with other congenital malformations, the most common being diaphragmatic hernia. 1 ^-^ 3

 This type of malformation may initially be identified on chest X-ray as a homogeneous opaque mass. Diagnosis is confirmed using chest CT, magnetic resonance imaging, or arteriography, the last of which is the best diagnostic method. 1 ^,^ 2

 The classic treatment for pulmonary sequestration is surgical resection of the sequestrated lobe or segment by thoracotomy or videothoracoscopy. 1 ^-^ 3 However, endovascular treatment is another option available nowadays; a less invasive procedure with a lower incidence of complications, since accidental transection of the artery supplying blood during surgical resection can cause massive hemorrhage with fatal consequences. 1 ^,^ 4

 This article describes a case of pulmonary sequestration and presents an endovascular method for treatment of the comorbidity. 

## CASE DESCRIPTION

 The patient was a 29-year-old female telemarketing operative, born and resident in São Paulo, Brazil, who was referred to a pulmonologist with a diagnosis of recurrent pneumonia. She reported symptoms of dyspnea in response to moderate effort, both during non-acute periods and during crises. Her previous medical history included episodes of recurrent pneumonia associated, initially, with dyspnea, coughing, chest pain, and fever from 5 years of age onwards. She had often presented at walk-in clinics, which treated her with antibiotics, achieving temporary resolution. 

**Figure 1:**
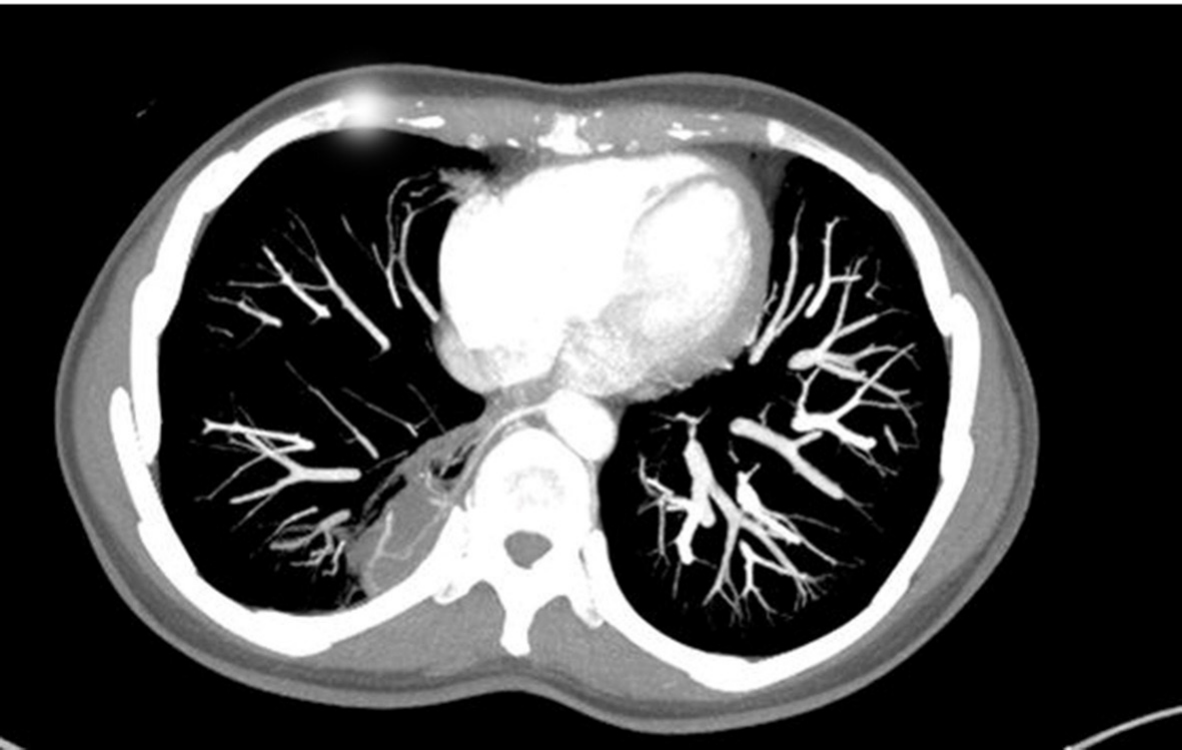
Axial tomography slice showing region of pulmonary sequestration involving the lower region of the right lung.

**Figure 2:**
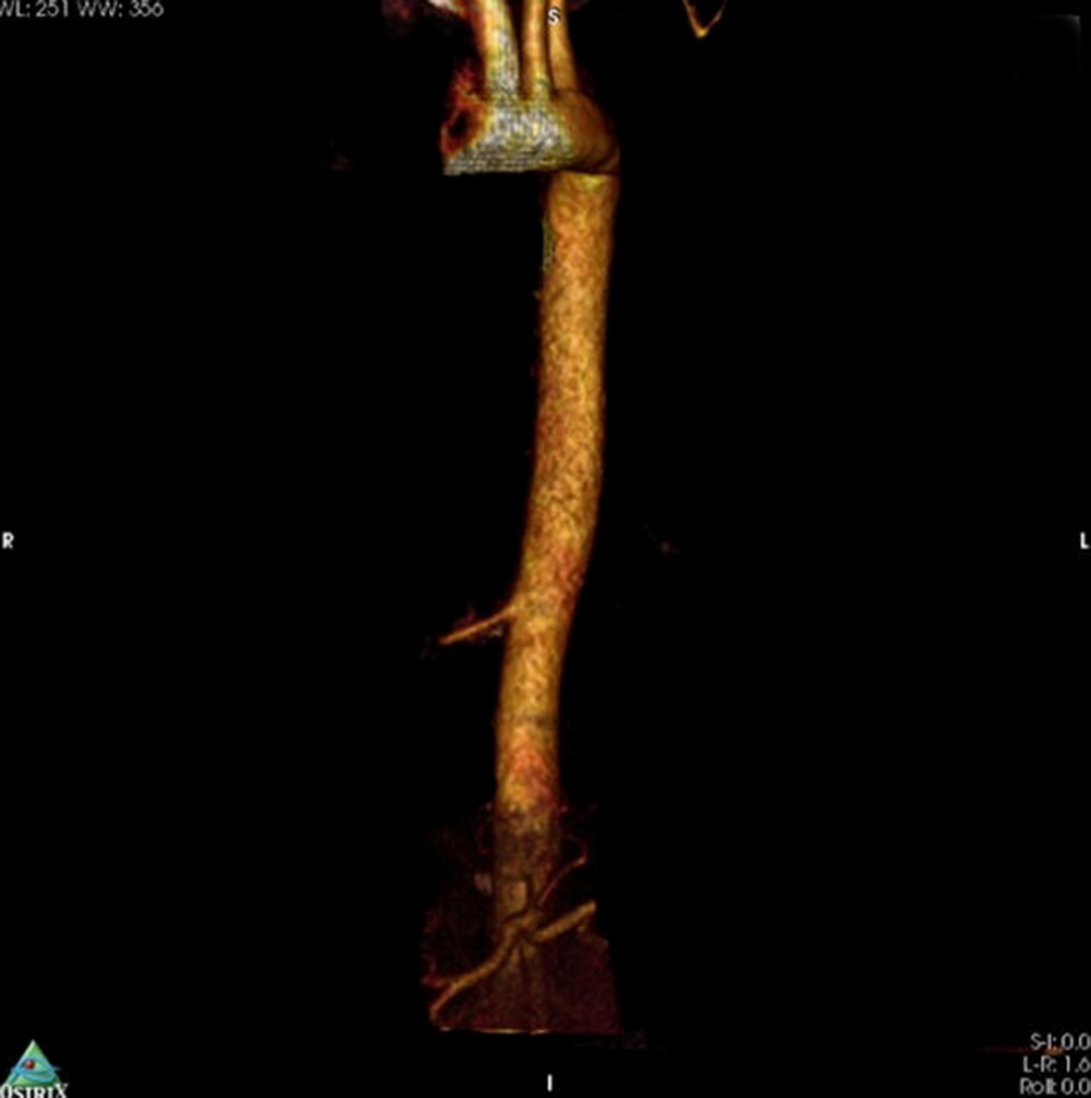
Angiotomography showing the aorta branch supplying the pulmonary sequestration.

 She stated that more recent crises had consisted of dyspnea only, with no fever, hemoptysis, chest pain, or coughing. She also stated that she did not smoke or drink. She was sedentary and her diet was regular. She denied any type of family history of pulmonary pathologies. Examinations requested as part of investigation included tomography with contrast and angiotomography ( Figures 1 and 2 ). Examination of tomography findings identified a vascular malformation originating in the aorta involving the lower region of the right lung. the patient was diagnosed with right pulmonary sequestration. The therapeutic management approach chosen was embolization of the anomalous vessel, for which the patient was referred to the vascular surgery service. 

**Figure 3:**
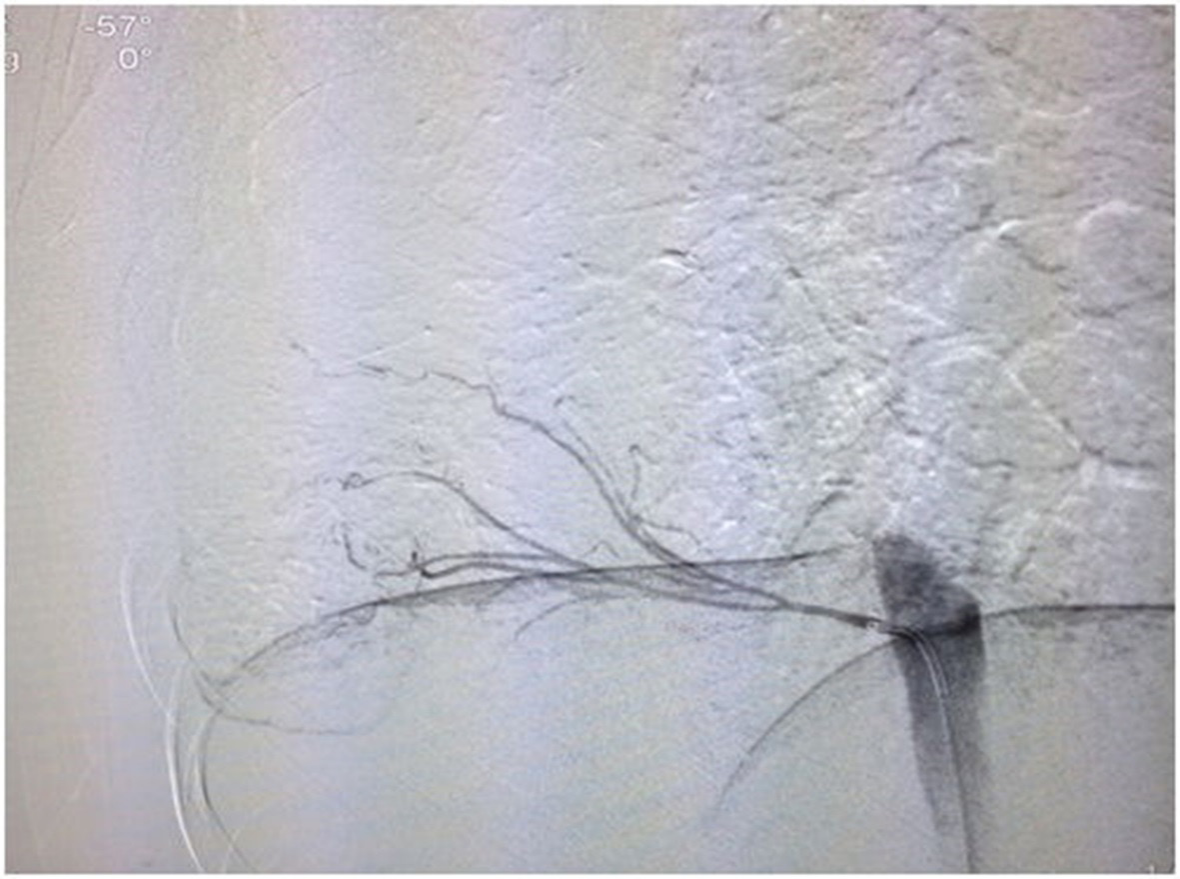
Angiography showing the anomalous branch of the aorta.

**Figure 4:**
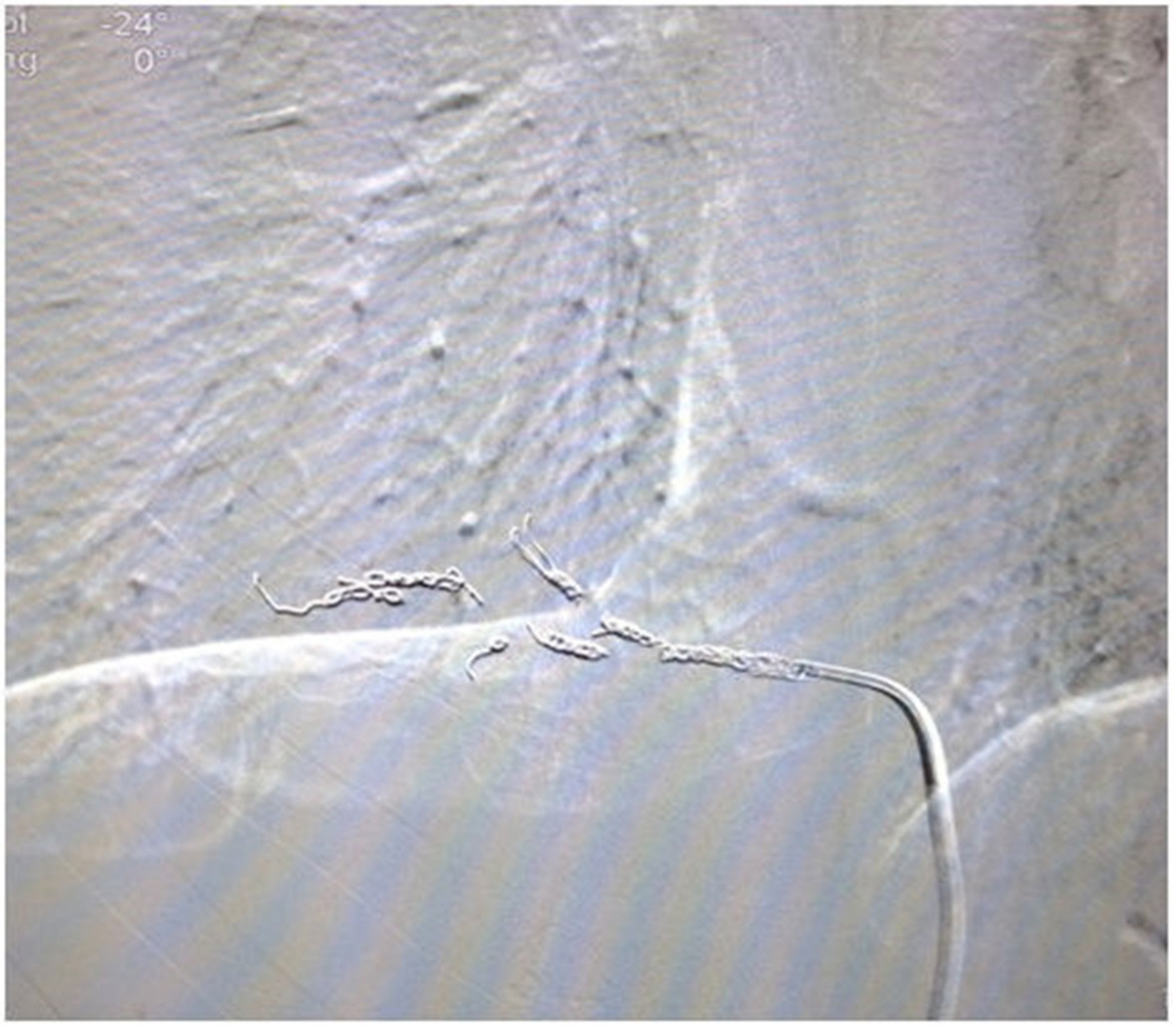
Embolization of the branch with controlled-release coils.

**Figure 5:**
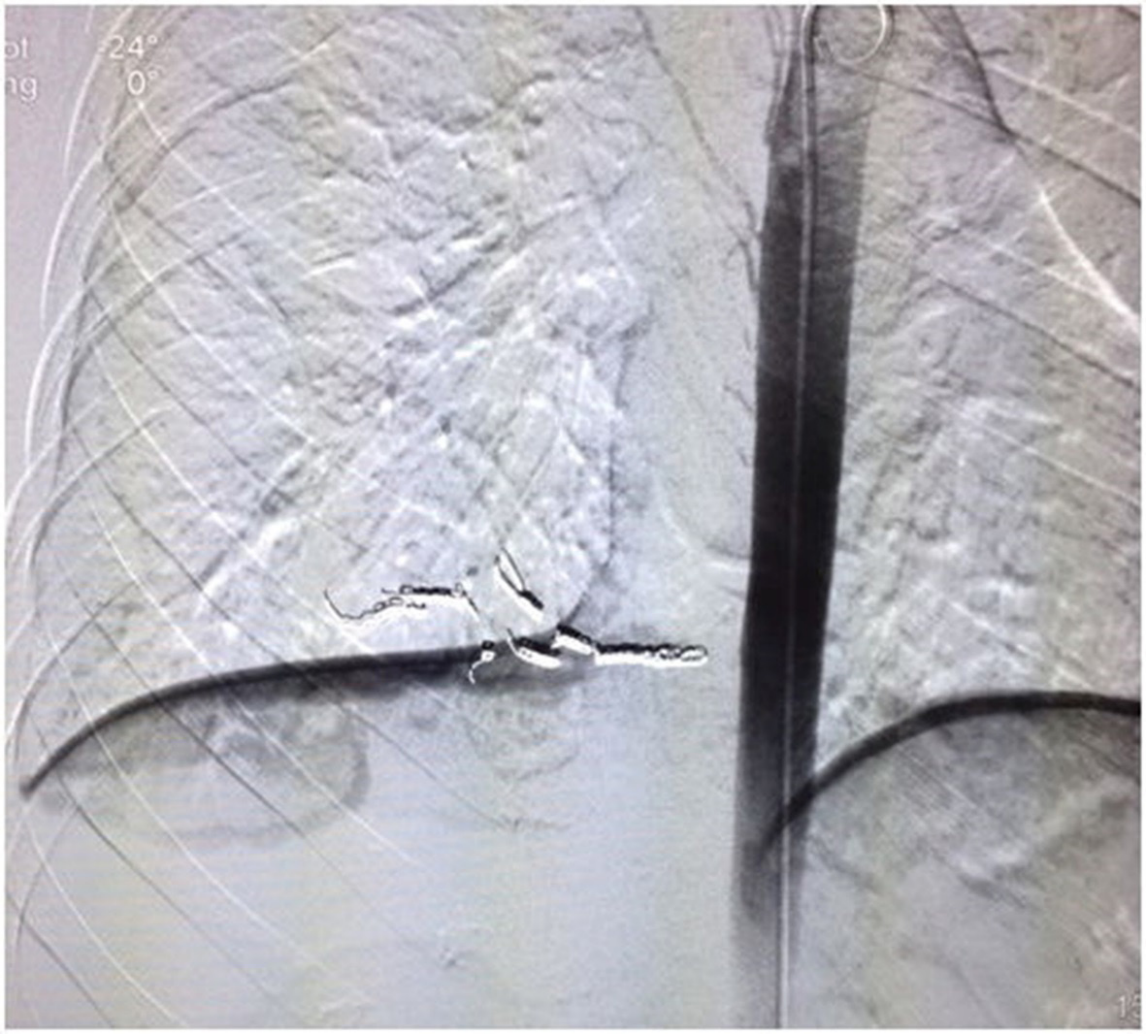
Angiography of the branch, normalized after embolization.

 Under local anesthesia with sedation, the patient was placed in dorsal decubitus. The right femoral artery was punctured, followed by catheterization of the anomalous artery branch ( Figure 3 ). A microcatheter was used to place eight controlled-release coils (Complex True Fill 3x10 and 4x10, Codman & Shurtleff, a Johnson & Johnson© franchise, Raynham, United States) into the branch to embolize it ( Figure 4 ). At the end of the surgical procedure ( Figure 5 ) and during the subsequent postoperative period, the patient remained free from any type of complication and did not need to be admitted to the intensive care unit. 

 The patient recovered well and was discharged at the end of the day after the operation. She is in outpatients follow-up with a pulmonologist and has been asymptomatic for 1 year, with no further pneumonia crises. 

## DISCUSSION

 The first description of an anomalous pulmonary artery deriving from the aorta was written by Hubber, in 1777. In 1861, Rokitansky and Rektorzik described cases that appear to have been extralobar pulmonary sequestration, but the term “sequestration” was only proposed by Pryce in 1946 and, since then, the finding has been recognized as a clinical entity. 5 ^,^ 6

 Two types of presentation of pulmonary sequestration are described in the literature: extralobar, in which there is total anatomic separation from the adjacent normal pulmonary parenchyma, and intralobar, in which the anomalous pulmonary segment is contiguous with the adjacent normal lung. 7 These malformation involve the lower lobes in more than 95% of cases and are more common on the left (55-60%). 8 The artery supplying the sequestration is generally a branch of the thoracic aorta and venous drainage is typically performed by pulmonary veins (95%). 8 This study reports a case of intralobar pulmonary sequestration in the lower right lung of a female patient, supplied by an anomalous branch of the thoracic aorta, diagnosed, and treated with embolization. 

 Pulmonary sequestration can be difficult to diagnose, since the symptoms it causes, such as dyspnea, respiratory infections, hemoptysis or, less frequently, chest pain, can be present in more common clinical conditions, including typical pneumonias or tuberculosis. The pathology may be identified in the prenatal period, during the second trimester, by examinations such as Doppler ultrasonography. 5 ^,^ 7 In these cases, it will frequently present as a solid hyperechogenic mass, which in the majority of cases is small with intense circulation in the interior. 5 ^,^ 7

 In children and adults, other imaging exams can be used, such as helical computed angiotomography of the chest, aortography and, if doubt remains, arteriography. It is worth pointing out that, although uncommon, the high blood flow from a vessel of the aorta may lead to an erroneous diagnosis of primary heart valve disease. The principal conditions to be ruled out during differential diagnosis include congenital or acquired pulmonary cyst, tumors of the posterior mediastinum, Bochdalek hiatus hernia, and congenital cyst of the diaphragm. 5 ^,^ 6

 The most common treatment for symptomatic cases of sequestration is surgical resection of the pulmonary parenchyma involved, whether lobe (lobectomy) or segment (segmentectomy). 1 ^-^ 3 ^,^ 5 ^-^ 8 These are highly invasive procedures and, furthermore, accidental transections can cause massive hemorrhage and death. 5 In the case of intralobar sequestration, in addition to the pathologic region sharing the same pleura as the normal lung remnant, it may also have inflammatory deformations from previous infections and thus make resection more difficult because of destruction of the intersegmental plane. 5 ^,^ 6 The most common complications of theses operations are hemothorax and empyema. 6

 An alternative treatment that has been described little in the literature, but which is increasingly popular in medical practice, is embolization of the anomalous vessel supplying the sequestrated pulmonary region, causing progressive infarction of the anomalous pulmonary tissue. Although this approach is still in development, it is a treatment option with promising characteristics in comparison with the surgical procedure described as ideal by the majority of authors. Surgery is an invasive process with a long recovery period and higher risk of infections, whereas embolization is minimally invasive, has a short recovery time, involves a lower risk of complications, and, as such, is less morbid. Certain effects, such as localized pain, nausea, and coughing may occur, but they are minimal when compared with the effects caused by surgery. In the case described here, we observed good results from this new procedure for patients with pulmonary sequestration. 

## References

[1] Westphal FL, Lima LC, Lima JC, Cardoso MS, Silva MS, Westphal DC (2012). Tumor carcinoide e sequestro pulmonar. J Bras Pneumol.

[2] Oliveira IM, Opaleye DT, Santiago JF (2008). Sequestro pulmonar extralobar: análise anatomopatológica de dois casos em natimortos e revisão da literatura. J Bras Patol Med Lab.

[3] Fiorotto WB, Zacarias L, Santos MR, Oliveira FB, Dib JE, Ramos GC (2012). Paciente com sequestro pulmonar intralobar: uma rara anomalia congênita. Rev Bras Cardiol Invasiva.

[4] Kanaan D, Motta CA, Coutinho AC (2015). Tratamento endovascular de hemoptise relacionada à sequestro pulmonar.

[5] Andrade LF, Haussmann MF, Coelho NM (2013). Sequestro pulmonar: revisão de literatura. Braz J Surg Clinical Res..

[6] Pêgo-Fernandes PM, Freire CH, Jatene FB, Beyruti R, Suso FV, Oliveira SA (2002). Sequestro pulmonar: uma série de nove casos operados. J Pneumol.

[7] Grossi S, Benigno B, Carvalho MV, Marchi E (2008). Sequestro pulmonar congênito: raro e letal. Perspectivas Médicas.

[8] Pinto DR, Avino AJ, Brandão SL (2009). Sequestro extralobar com hemotórax secundário a infarto pulmonar. J Bras Pneumol.

