# Supplementary material for: Pulmonary sequestration and endovascular treatment: a case report
Source: J Vasc Bras. 2019 Jan 10;18:e20180110. doi: 10.1590/1677-5449.011018 (PMC6542322; doi:10.1590/1677-5449.011018)
Supplement: Translation

# Translation: Sequestro pulmonar e tratamento endovascular: um relato de caso

**Authors:** Sergio Quilici Belczak, Ingredy Tavares da Silva, Jéssica Cunha Bernardes, Felipe Basso de Macedo, Laís Leite Lucato, Bruna Rodrigues, Bruna Stecca Zeque

**Affiliations:** 1 Centro Universitário São Camilo, São Paulo, SP, Brasil.; 2 Instituto de Aprimoramento e Pesquisa em Angiorradiologia e Cirurgia Endovascular – IAPACE, São Paulo, SP, Brasil.

**Keywords:** sequestro pulmonar, embolização, circulação pulmonar, procedimentos endovasculares

## Abstract

O sequestro pulmonar refere-se à anomalia congênita definida por massa de parênquima pulmonar não funcionante. É dividido pela existência de envoltório pleural próprio em intralobar, representando cerca de 75% dos casos, ou extralobar, responsável pelos 25% restantes. O diagnóstico é feito através de radiografia e confirmado com tomografia computadorizada, ressonância nuclear magnética e arteriografia. Tradicionalmente, o tratamento proposto é cirúrgico, mas a técnica endovascular tem apresentado bons resultados. É relatado o caso de uma mulher, 29 anos, com quadro clínico de pneumonias de repetição desde os 5 anos. A tomografia de tórax revelou malformação vascular em região inferior de pulmão direito. O tratamento vascular foi realizado através de embolização do ramo anômalo.

## INTRODUÇÃO

 As malformações pulmonares, embora classificadas em patologias distintas, formam um conjunto de anomalias que possuem manifestações clínicas muito semelhantes e que se originam a partir de falhas no desenvolvimento embrionário. Entre as malformações mais comuns, podemos citar a malformação adenomatoide cística, o sequestro pulmonar, o enfisema lobar congênito, os cistos broncogênicos e as malformações arteriovenosas pulmonares 1 . Entre essas entidades, o sequestro pulmonar corresponde a 0,15 a 6,45% dos casos de malformação pulmonar 1 ^-^ 3 . 

 O sequestro pulmonar é, portanto, uma anomalia congênita rara. Trata-se de uma massa de parênquima pulmonar não funcionante, sem comunicação com a árvore traqueobrônquica normal e que possui o suprimento sanguíneo advindo de uma artéria sistêmica anômala, sendo realizado, geralmente, pela artéria aorta descendente, embora possa haver outras fontes de suprimento arterial. Já sua drenagem pode ser realizada tanto por veias sistêmicas quanto por veias pulmonares 1 ^-^ 3 . Esse tecido é composto por tecido embrionário cístico e alvéolos não aerados e desorganizados, além de outros componentes do trato respiratório 1 . 

 Essa malformação pode ser classificada em duas formas distintas, dependendo da existência de um envoltório pleural próprio, denominadas intra e extralobar 1 ^-^ 3 . O sequestro pulmonar intralobar ocorre quando a área sequestrada se apresenta revestida por pleura visceral de um lobo pulmonar normal 1 ^-^ 3 . Essa forma de sequestro corresponde a aproximadamente 75% dos casos, sendo mais comum nos lobos inferiores à esquerda, com igual distribuição em relação ao sexo. A forma intralobar costuma apresentar manifestações clínicas mais tardias, por volta da segunda década de vida, geralmente por infecções respiratórias recorrentes, hemoptise e dispneia 1 ^,^ 3 . 

 Já o sequestro pulmonar extralobar ocorre quando a região sequestrada está revestida por um envoltório pleural próprio, representando 25% dos casos desse tipo de malformação pulmonar 1 ^-^ 3 . Essa forma também ocorre, predominantemente, nos lobos pulmonares inferiores à esquerda, mas, diferentemente do que ocorre na entidade intralobar, apresenta maior prevalência no sexo masculino (4:1) 2 ^,^ 3 . A apresentação clínica costuma ocorrer já nos primeiros meses de vida e está frequentemente associada a outras malformações congênitas, sendo a mais comum a hérnia diafragmática 1 ^-^ 3 . 

 Esse tipo de malformação pode ser inicialmente identificado na radiografia de tórax como uma massa de opacidade homogênea. A confirmação diagnóstica é realizada com tomografia de tórax, ressonância nuclear magnética ou arteriografia, sendo este último o melhor método diagnóstico 1 ^,^ 2 . 

 O tratamento clássico do sequestro pulmonar é a ressecção cirúrgica do lobo ou do segmento sequestrado por toracotomia ou videotoracoscopia 1 ^-^ 3 . Contudo, atualmente existe a possibilidade do tratamento endovascular, um procedimento menos invasivo e com menor incidência de complicações, já que a transecção acidental da artéria nutriz durante a ressecção cirúrgica pode causar hemorragia maciça com consequências fatais 1 ^,^ 4 . 

 O presente trabalho relata um caso de sequestro pulmonar e apresenta o método endovascular como forma de tratamento para tal comorbidade. 

## DESCRIÇÃO DO CASO

 Paciente do sexo feminino, 29 anos, operadora de telemarketing, natural e procedente de São Paulo, foi encaminhada ao pneumologista com diagnóstico de pneumonia de repetição. Referiu sintomatologia de dispneia por esforços moderados tanto no período intercrítico quanto durante as crises. Na história médica pregressa, a paciente relatou episódios de pneumonia de repetição associada, inicialmente, a dispneia, tosse, dor torácica e febre desde os 5 anos de idade. A paciente procurou diversas vezes o pronto-atendimento, e a conduta clínica foi antibioticoterapia com resolução temporária dos eventos. 

 A paciente referiu que as crises tardias apresentaram apenas dispneia, negando febre, hemoptise, dor torácica e tosse. Negou tabagismo e etilismo. Apresentou-se sedentária e com alimentação regular. Como antecedentes familiares, negou qualquer patologia pulmonar. Foram solicitados exames complementares para ajudar a elucidar o caso, incluindo tomografia contrastada e angiotomografia ( Figuras 1 e 2 ). Após pesquisa com tomografia, identificou-se a presença de uma malformação vascular originada da aorta na região inferior do pulmão direito. Foi diagnosticado, então, um sequestro pulmonar à direita. Quanto à conduta terapêutica, foi escolhida a embolização do vaso anômalo e, para isso, a paciente foi encaminhada ao serviço de cirurgia vascular. 

**Figura 1:** Tomografia em corte axial em que se identifica área de sequestro pulmonar na região inferior do pulmão direito.

**Figura 2:** Angiotomografia evidenciando ramo aórtico promovendo o sequestro pulmonar.

 Após ser submetida a anestesia local com sedação, a paciente foi posicionada em decúbito dorsal. Realizou-se punção da artéria femoral direita e cateterização do ramo anômalo ( Figura 3 ). Nesse ramo, mediante a utilização de microcateter, foram introduzidas oito molas de liberação controlada (Complex True Fill 3x10 e 4x10), fabricadas pela Codman & Shurtleff, franquia da Johnson & Johnson^©^, procedente de Raynham, Estados Unidos da América, para embolização do ramo ( Figura 4 ). Ao término do procedimento cirúrgico ( Figura 5 ), bem como no pós-operatório subsequente, a paciente não apresentou qualquer intercorrência e não houve necessidade de internação na unidade de terapia intensiva. 

**Figura 3:** Angiografia evidenciando ramo anômalo proveniente da aorta.

**Figura 4:** Embolização do ramo com molas de liberação controlada.

**Figura 5:** Angiografia do ramo normalizado após embolização.

 A paciente apresentou boa evolução pós-operatória e recebeu alta ao término do primeiro dia do pós-operatório. Encontra-se em acompanhamento ambulatorial com o pneumologista e está assintomática há um ano, sem novas crises de pneumonia. 

## DISCUSSÃO

 A primeira descrição de uma artéria anômala pulmonar derivada da aorta foi feita por Hubber, em 1777. Em 1861, Rokitansky e Rektorzik relataram casos que pareciam ser de sequestro pulmonar extralobar, mas somente em 1946 o termo “sequestro” foi proposto por Pryce e, a partir de então, esse achado foi reconhecido como uma entidade clínica 5 ^,^ 6 . 

 São retratados na literatura dois tipos de apresentação do sequestro pulmonar: o extralobar, no qual se mantém uma completa separação anatômica do parênquima pulmonar normal adjacente, e o intralobar, no qual o segmento pulmonar anômalo é contíguo ao pulmão normal adjacente 7 . A malformação afeta os lobos inferiores em mais de 95% dos casos e, mais comumente, à esquerda (55-60%) 8 . A artéria que irriga o sequestro geralmente é ramo da aorta torácica, enquanto a drenagem venosa tipicamente é realizada pelas veias pulmonares (95%) 8 . Neste estudo, relata-se um caso de sequestro pulmonar intralobar localizado no lobo inferior do pulmão direito da paciente e proveniente de um ramo anômalo da aorta torácica, diagnosticado e tratado através de embolização. 

 O diagnóstico do sequestro pulmonar pode ser difícil, uma vez que os sintomas originados pelo distúrbio, como dispneia, infecções respiratórias, hemoptise ou, menos frequentemente, dor no peito, podem estar presentes em situações clínicas mais comuns, incluindo pneumonias típicas ou tuberculose. A identificação da patologia pode ser feita no período pré-natal, durante o segundo trimestre, por meio de exames como a ultrassonografia com Doppler 5 ^,^ 7 . Nesses casos, apresenta-se mais frequentemente sob forma de uma massa sólida hiperecogênica, na maioria das vezes pequena e com intensa circulação em seu interior 5 ^,^ 7 . 

 Já em crianças e adultos outros exames de imagem podem ser usados, tais como angiotomografia helicoidal de tórax, aortografia e, em caso de dúvida persistente, arteriografia. Vale ressaltar que apesar de pouco comum, o elevado fluxo sanguíneo oriundo de um vaso da aorta pode propiciar o diagnóstico errôneo de doença valvular cardíaca primária. Entre as principais afecções a serem consideradas como diagnóstico diferencial estão cisto pulmonar congênito ou adquirido, tumores de mediastino posterior, hérnia hiatal de Bochdalek e cisto diafragmático congênito 5 ^,^ 6 . 

 A terapêutica mais utilizada para os casos sintomáticos de sequestro é a ressecção cirúrgica do parênquima pulmonar acometido, seja ele um lobo (lobectomia) ou segmento (segmentectomia) 1 ^-^ 3 ^,^ 5 ^-^ 8 . Tal procedimento é consideravelmente invasivo; somadas a isso, transecções acidentais podem levar a hemorragia maciça e óbito 5 . No caso do sequestro intralobar, a região patológica, além de dividir a mesma pleura com o restante do pulmão normal, pode também possuir deformações inflamatórias decorrentes de infecções prévias e, dessa forma, complicar a ressecção por conta da destruição do plano intersegmental 5 ^,^ 6 . As principais complicações operatórias são hemotórax e empiema 6 . 

 Outro tratamento pouco descrito na literatura, mas que vem ganhando espaço na prática médica é a embolização do vaso anômalo, provedor da área pulmonar sequestrada, que ocasiona infarto progressivo do tecido pulmonar anômalo. Apesar de ser uma abordagem ainda em desenvolvimento, essa terapêutica possui características promissoras em relação ao procedimento cirúrgico, retratado como ideal pela maioria dos autores. Enquanto a cirurgia é um processo invasivo com maior período de recuperação e maior risco de infecções, a embolização é minimamente invasiva, propicia um curto período de recuperação e envolve menor risco de complicações, sendo assim menos mórbida. Alguns efeitos como dores locais, náuseas e tosse podem aparecer, mas são mínimos quando comparados aos efeitos causados por uma cirurgia. Através do caso relatado, notamos os bons resultados desse novo procedimento em pacientes que apresentam sequestro pulmonar.
